# *N*-(3-Methoxy­phen­yl)-*tert*-butane­sulfinamide

**DOI:** 10.1107/S1600536809052507

**Published:** 2009-12-12

**Authors:** Mrityunjoy Datta, Alan J. Buglass, Mark R. J. Elsegood

**Affiliations:** a Department of Chemistry, KAIST, Daejeon 305-701, Republic of Korea; b Chemistry Department, Loughborough University, Loughborough LE11 3TU, England

## Abstract

In the title compound, C_11_H_17_NO_2_S, the mol­ecules inter­act in a head-to-tail fashion through pairs of N—H⋯O hydrogen bonds, giving discrete centrosymmetric dimers. The N(H)S(O)^*t*^Bu fragment is disordered over two sets of positions, with the major component comprising 90.0 (2)%.

## Related literature

For *N*-aryl­alkanesulfinamides, see: Datta *et al.* (2008 ▶, 2009 ▶). For *N*-alkyl­alkanesulfinamides, see: Sato *et al.* (1975 ▶); Ferreira *et al.* (2005 ▶); Schuckmann *et al.* (1978 ▶). For the synthesis, see: Stretter *et al.* (1969 ▶).
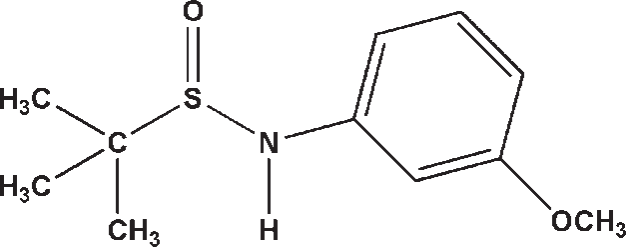


## Experimental

### 

#### Crystal data

C_11_H_17_NO_2_S
*M* *_r_* = 227.32
Monoclinic, 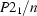

*a* = 12.4068 (13) Å
*b* = 7.3076 (8) Å
*c* = 12.9230 (13) Å
β = 93.2992 (15)°
*V* = 1169.7 (2) Å^3^
*Z* = 4
Mo *K*α radiation
μ = 0.26 mm^−1^
*T* = 150 K
0.37 × 0.22 × 0.20 mm

#### Data collection

Bruker APEXII CCD diffractometer
Absorption correction: multi-scan (*SADABS*; Sheldrick, 2007 ▶) *T*_min_ = 0.911, *T*_max_ = 0.950
10627 measured reflections
2633 independent reflections
2237 reflections with *I* > 2σ(*I*)
*R*_int_ = 0.032

#### Refinement

*R*[*F* ^2^ > 2σ(*F* ^2^)] = 0.042
*wR*(*F* ^2^) = 0.117
*S* = 1.07
2633 reflections
166 parameters
149 restraints
H-atom parameters constrained
Δρ_max_ = 0.62 e Å^−3^
Δρ_min_ = −0.40 e Å^−3^

### 

Data collection: *APEX2* (Bruker, 2006 ▶); cell refinement: *SAINT* (Bruker, 2006 ▶); data reduction: *SAINT*; program(s) used to solve structure: *SHELXS97* (Sheldrick, 2008 ▶); program(s) used to refine structure: *SHELXL97* (Sheldrick, 2008 ▶); molecular graphics: *SHELXTL* (Sheldrick, 2008 ▶); software used to prepare material for publication: *SHELXTL* and local programs.

## Supplementary Material

Crystal structure: contains datablocks I, global. DOI: 10.1107/S1600536809052507/ng2698sup1.cif

Structure factors: contains datablocks I. DOI: 10.1107/S1600536809052507/ng2698Isup2.hkl

Additional supplementary materials:  crystallographic information; 3D view; checkCIF report

## Figures and Tables

**Table 1:** Hydrogen-bond geometry (Å, °)

*D*—H⋯*A*	*D*—H	H⋯*A*	*D*⋯*A*	*D*—H⋯*A*
N1—H1⋯O1^i^	0.88	2.24	2.884 (2)	130
N1*X*—H1*X*⋯O1*X*^ii^	0.88	2.21	2.94 (2)	141

Symmetry codes: (i) 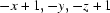; (ii) 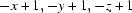.

**Fig. 1.**
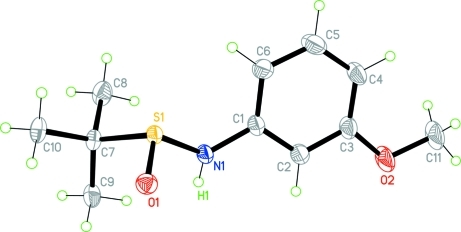
Molecular structure of the major component of (I) with atom labels and 50% probability displacement ellipsoids for non-H atoms.

**Fig. 2.**
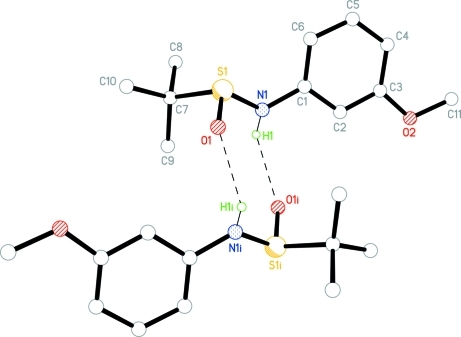
Centrosymmetric dimer of (I) in the crystal packing of the major component, showing intermolecular hydrogen bonding. Symmetry code i = -x + 1, -y, -z + 1.

## supplementary crystallographic information

### Comment

The molecular structure of the title compound (I) exhibits disorder of the N(H)S(O)*^t^*Bu fragment over two sets of positions. The major component comprises 90.0 (2)% (Fig. 1), in which the *N*—C_aryl _bond length is 1.418 (2) Å, similar to that [1.4225 (14) Å] in *N*-(4-methoxyphenyl)-*tert*-butanesulfinamide (Datta *et al.*, 2009). The corresponding bond length [1.457 (16) Å] in the minor component is longer, though far less precisely determined. This perhaps suggests weaker delocalization of electrons over N and the aromatic ring, which would correlate with the greater non-coplanarity of the aromatic ring and sulfinyl moiety in this component. In either case, however, the *N*—C_aryl _bond is shorter than the *N*—C_alkyl_ bonds [1.470–1.530 Å] observed in structures of *N*-alkylalkanesulfinamides (Sato *et al.*, 1975; Schuckmann *et al.*, 1978; Ferreira *et al.*, 2005). The crystal packing of (I) shows head-to-tail interaction through NH···OS hydrogen bonds, forming discrete centrosymmetric dimers, as illustrated for the major component in Fig. 2. The hydrogen bonding data for both components are listed in Table 1. There is no evidence of hydrogen bonding involving the methoxy group, nor of weak CH···.OS hydrogen bonding, as observed in the packing of *N*-phenyladamantane-1-sulfinamide (Datta *et al.*, 2008).

### Experimental

Compound (I) was prepared by the method of Stretter *et al.* (1969), using *tert*-butanesulfinyl chloride (281 mg, 2 mmol) and 3-methoxyaniline (492 mg, 4 mmol) in dry ether (20 ml). After 5 h (with TLC monitoring) the colourless solid amine salt was fitered off and the solvent was removed under reduced pressure. Column chromatography (silica gel, 1% methanol-dichloromethane) provided (I) as colourless crystals (430 mg, 95%), m.p. 367 K. Single crystals suitable for X-ray analysis were obtained by evaporation of a solution of (I) in dichloromethane:hexane (1:1) at room temperature. Spectroscopic analysis: FTIR (KBr) (cm^-1^) 3024, 1603, 1496, 1473, 1368, 1278, 1227, 1214, 1156, 1069, 953, 834. ^1^H NMR (400 MHz, CDCl_3_ p.p.m. with respect to TMS) δ 7.13 (dd, J = 8.0, 8.5 Hz, 1H), 6.58–6.53 (m, 3H), 5.41 (bs, 1H), 3.75 (d, J = 0.6 Hz, 3H), 1.30 (s, 9H). ^13^C (100 MHz, CDCl_3_ p.p.m. with respect to TMS) δ 160.6, 143.3, 130.2, 110.6, 108.4, 104.2, 56.4. 55.2. 22.4. EIMS m/*z* (%) 228 (*MH^+^*, 85), 227 (*M^+^*, 25), 213 (17), 171 (*MH^+^*- tBu, 100), 123 (*MH^+^* - *^t^*BuSO, 6), 108 (*MH^+^* - *^t^*BuSONH, 12), 95 (53). To our knowledge, these are the first reported analytical data for (I).

### Refinement

H atoms were located in a difference Fourier map and refined geometrically using a riding model. Methyl groups were refined with rotational freedom. Lengths and displacement parameters were constrained as follows: C—H = 0.95–0.98 Å and *U_iso_*(H) = 1.2 (1.5 for CH_3_) times *U_eq_*(C, N). The minor disorder component was refined isotropically. The disorder was modelled with the aid of geometrical and displacement parameter restraints.

### Crystal data

**Table:**

C_11_H_17_NO_2_S	*F*(000) = 488
*M**_r_* = 227.32	*D*_x_ = 1.291 Mg m^−^^3^
Monoclinic, *P*2_1_/*n*	Mo *K*α radiation, λ = 0.71073 Å
Hall symbol: -P 2yn	Cell parameters from 4390 reflections
*a* = 12.4068 (13) Å	θ = 2.2–27.2°
*b* = 7.3076 (8) Å	µ = 0.26 mm^−^^1^
*c* = 12.9230 (13) Å	*T* = 150 K
β = 93.2992 (15)°	Block, colourless
*V* = 1169.7 (2) Å^3^	0.37 × 0.22 × 0.20 mm
*Z* = 4	

### Data collection

**Table:**

Bruker APEXII CCD diffractometer	2633 independent reflections
Radiation source: fine-focus sealed tube	2237 reflections with *I* > 2σ(*I*)
graphite	*R*_int_ = 0.032
ω rotation with narrow frames scans	θ_max_ = 27.3°, θ_min_ = 2.2°
Absorption correction: multi-scan (*SADABS*; Sheldrick, 2007)	*h* = −15→16
*T*_min_ = 0.911, *T*_max_ = 0.950	*k* = −9→9
10627 measured reflections	*l* = −16→16

### Refinement

**Table:**

Refinement on *F*^2^	Primary atom site location: structure-invariant direct methods
Least-squares matrix: full	Secondary atom site location: difference Fourier map
*R*[*F*^2^ > 2σ(*F*^2^)] = 0.042	Hydrogen site location: inferred from neighbouring sites
*wR*(*F*^2^) = 0.117	H-atom parameters constrained
*S* = 1.07	*w* = 1/[σ^2^(*F*_o_^2^) + (0.056*P*)^2^ + 0.7085*P*] where *P* = (*F*_o_^2^ + 2*F*_c_^2^)/3
2633 reflections	(Δ/σ)_max_ = 0.001
166 parameters	Δρ_max_ = 0.62 e Å^−^^3^
149 restraints	Δρ_min_ = −0.40 e Å^−^^3^

### Special details

**Table:**

Geometry. All e.s.d.'s (except the e.s.d. in the dihedral angle between two l.s. planes) are estimated using the full covariance matrix. The cell e.s.d.'s are taken into account individually in the estimation of e.s.d.'s in distances, angles and torsion angles; correlations between e.s.d.'s in cell parameters are only used when they are defined by crystal symmetry. An approximate (isotropic) treatment of cell e.s.d.'s is used for estimating e.s.d.'s involving l.s. planes.
Refinement. Refinement of *F*^2^ against ALL reflections. The weighted *R*-factor *wR* and goodness of fit *S* are based on *F*^2^, conventional *R*-factors *R* are based on *F*, with *F* set to zero for negative *F*^2^. The threshold expression of *F*^2^ > σ(*F*^2^) is used only for calculating *R*-factors(gt) *etc*. and is not relevant to the choice of reflections for refinement. *R*-factors based on *F*^2^ are statistically about twice as large as those based on *F*, and *R*- factors based on ALL data will be even larger.

### Fractional atomic coordinates and isotropic or equivalent isotropic displacement parameters (Å^2^)

**Table:**

	*x*	*y*	*z*	*U*_iso_*/*U*_eq_	Occ. (<1)
O1	0.54940 (10)	−0.05258 (18)	0.36978 (10)	0.0229 (3)	0.900 (2)
S1	0.50816 (3)	0.13248 (6)	0.33748 (3)	0.01938 (15)	0.900 (2)
N1	0.42312 (12)	0.2035 (3)	0.42415 (12)	0.0236 (4)	0.900 (2)
H1	0.4482	0.2281	0.4877	0.028*	0.900 (2)
C1	0.31128 (13)	0.2253 (2)	0.39789 (13)	0.0253 (4)	
C2	0.24035 (14)	0.2103 (3)	0.47687 (14)	0.0277 (4)	
H2	0.2669	0.1811	0.5453	0.033*	
C3	0.13034 (14)	0.2383 (3)	0.45550 (14)	0.0289 (4)	
O2	0.06872 (11)	0.2258 (3)	0.54029 (11)	0.0462 (4)	
C11	−0.04594 (16)	0.2441 (4)	0.52212 (19)	0.0487 (6)	
H11A	−0.0626	0.3643	0.4916	0.073*	
H11B	−0.0808	0.2325	0.5879	0.073*	
H11C	−0.0727	0.1480	0.4744	0.073*	
C4	0.08935 (14)	0.2716 (3)	0.35566 (15)	0.0300 (4)	
H4	0.0140	0.2867	0.3407	0.036*	
C5	0.16122 (16)	0.2821 (3)	0.27816 (15)	0.0384 (5)	
H5	0.1340	0.3040	0.2090	0.046*	
C6	0.27117 (15)	0.2620 (3)	0.29758 (14)	0.0328 (4)	
H6	0.3188	0.2731	0.2429	0.039*	
C7	0.62017 (14)	0.2928 (3)	0.36663 (14)	0.0212 (4)	0.900 (2)
C8	0.57870 (17)	0.4793 (3)	0.32914 (18)	0.0314 (5)	0.900 (2)
H8A	0.5209	0.5198	0.3724	0.047*	0.900 (2)
H8B	0.5507	0.4698	0.2569	0.047*	0.900 (2)
H8C	0.6379	0.5682	0.3341	0.047*	0.900 (2)
C9	0.65533 (15)	0.2918 (3)	0.48138 (15)	0.0262 (4)	0.900 (2)
H9A	0.6810	0.1694	0.5015	0.039*	0.900 (2)
H9B	0.5939	0.3248	0.5220	0.039*	0.900 (2)
H9C	0.7137	0.3807	0.4945	0.039*	0.900 (2)
C10	0.7108 (2)	0.2247 (6)	0.3005 (2)	0.0282 (7)	0.900 (2)
H10A	0.7359	0.1048	0.3258	0.042*	0.900 (2)
H10B	0.7710	0.3118	0.3054	0.042*	0.900 (2)
H10C	0.6835	0.2141	0.2281	0.042*	0.900 (2)
O1X	0.5509 (11)	0.5520 (16)	0.3695 (10)	0.032 (3)*	0.100 (2)
S1X	0.5084 (4)	0.3681 (6)	0.3374 (3)	0.0303 (15)*	0.100 (2)
N1X	0.4214 (12)	0.288 (2)	0.4216 (13)	0.026 (4)*	0.100 (2)
H1X	0.4450	0.2833	0.4871	0.032*	0.100 (2)
C7X	0.6173 (12)	0.204 (2)	0.3663 (12)	0.029 (4)*	0.100 (2)
C8X	0.5762 (16)	0.016 (2)	0.3308 (16)	0.034 (4)*	0.100 (2)
H8D	0.5135	−0.0179	0.3694	0.052*	0.100 (2)
H8E	0.6335	−0.0751	0.3436	0.052*	0.100 (2)
H8F	0.5553	0.0197	0.2565	0.052*	0.100 (2)
C9X	0.6514 (16)	0.199 (3)	0.4840 (12)	0.032 (5)*	0.100 (2)
H9D	0.6766	0.3204	0.5065	0.048*	0.100 (2)
H9E	0.7096	0.1097	0.4964	0.048*	0.100 (2)
H9F	0.5893	0.1635	0.5231	0.048*	0.100 (2)
C10X	0.7138 (19)	0.269 (4)	0.308 (2)	0.025 (8)*	0.100 (2)
H10D	0.7374	0.3893	0.3344	0.038*	0.100 (2)
H10E	0.6927	0.2793	0.2338	0.038*	0.100 (2)
H10F	0.7731	0.1813	0.3179	0.038*	0.100 (2)

### Atomic displacement parameters (Å^2^)

**Table:**

	*U*^11^	*U*^22^	*U*^33^	*U*^12^	*U*^13^	*U*^23^
O1	0.0234 (6)	0.0218 (7)	0.0233 (6)	0.0001 (5)	0.0005 (5)	0.0005 (5)
S1	0.0152 (2)	0.0251 (2)	0.0179 (2)	0.00068 (16)	0.00179 (14)	0.00043 (17)
N1	0.0151 (8)	0.0366 (11)	0.0193 (8)	0.0024 (7)	0.0019 (6)	0.0015 (7)
C1	0.0168 (8)	0.0337 (10)	0.0254 (9)	0.0007 (6)	0.0014 (6)	0.0012 (7)
C2	0.0215 (9)	0.0385 (10)	0.0230 (8)	−0.0003 (7)	0.0009 (6)	0.0003 (7)
C3	0.0189 (8)	0.0400 (10)	0.0282 (9)	0.0001 (7)	0.0047 (7)	−0.0026 (7)
O2	0.0164 (7)	0.0900 (13)	0.0327 (8)	0.0034 (7)	0.0060 (5)	0.0010 (7)
C11	0.0156 (9)	0.0850 (19)	0.0464 (12)	0.0009 (10)	0.0083 (8)	−0.0025 (12)
C4	0.0180 (8)	0.0380 (10)	0.0336 (10)	0.0047 (7)	−0.0021 (7)	−0.0017 (8)
C5	0.0284 (10)	0.0603 (14)	0.0259 (9)	0.0128 (9)	−0.0022 (7)	0.0034 (9)
C6	0.0243 (9)	0.0504 (12)	0.0241 (9)	0.0076 (8)	0.0050 (7)	0.0068 (8)
C7	0.0165 (8)	0.0226 (10)	0.0251 (9)	−0.0010 (7)	0.0064 (6)	−0.0021 (8)
C8	0.0282 (10)	0.0240 (10)	0.0431 (12)	0.0031 (8)	0.0109 (9)	0.0028 (9)
C9	0.0187 (9)	0.0324 (12)	0.0277 (10)	−0.0042 (8)	0.0026 (7)	−0.0061 (8)
C10	0.0195 (12)	0.0340 (18)	0.0321 (14)	0.0012 (10)	0.0109 (8)	−0.0028 (13)

### Geometric parameters (Å, °)

**Table:**

O1—S1	1.4967 (14)	C8—H8B	0.9800
S1—N1	1.6652 (16)	C8—H8C	0.9800
S1—C7	1.8401 (19)	C9—H9A	0.9800
N1—C1	1.418 (2)	C9—H9B	0.9800
N1—H1	0.8800	C9—H9C	0.9800
C1—C6	1.388 (3)	C10—H10A	0.9800
C1—C2	1.390 (2)	C10—H10B	0.9800
C1—N1X	1.457 (16)	C10—H10C	0.9800
C2—C3	1.392 (2)	O1X—S1X	1.493 (12)
C2—H2	0.9500	S1X—N1X	1.681 (13)
C3—O2	1.375 (2)	S1X—C7X	1.828 (13)
C3—C4	1.381 (3)	N1X—H1X	0.8800
O2—C11	1.435 (2)	C7X—C8X	1.528 (16)
C11—H11A	0.9800	C7X—C10X	1.528 (16)
C11—H11B	0.9800	C7X—C9X	1.555 (15)
C11—H11C	0.9800	C8X—H8D	0.9800
C4—C5	1.381 (3)	C8X—H8E	0.9800
C4—H4	0.9500	C8X—H8F	0.9800
C5—C6	1.381 (3)	C9X—H9D	0.9800
C5—H5	0.9500	C9X—H9E	0.9800
C6—H6	0.9500	C9X—H9F	0.9800
C7—C9	1.522 (3)	C10X—H10D	0.9800
C7—C8	1.526 (3)	C10X—H10E	0.9800
C7—C10	1.534 (3)	C10X—H10F	0.9800
C8—H8A	0.9800		
			
O1—S1—N1	108.28 (9)	H8B—C8—H8C	109.5
O1—S1—C7	106.04 (8)	C7—C9—H9A	109.5
N1—S1—C7	99.44 (9)	C7—C9—H9B	109.5
C1—N1—S1	121.54 (13)	H9A—C9—H9B	109.5
C1—N1—H1	119.2	C7—C9—H9C	109.5
S1—N1—H1	119.2	H9A—C9—H9C	109.5
C6—C1—C2	119.54 (16)	H9B—C9—H9C	109.5
C6—C1—N1	122.58 (16)	C7—C10—H10A	109.5
C2—C1—N1	117.88 (15)	C7—C10—H10B	109.5
C6—C1—N1X	114.7 (6)	H10A—C10—H10B	109.5
C2—C1—N1X	119.7 (6)	C7—C10—H10C	109.5
C1—C2—C3	119.86 (16)	H10A—C10—H10C	109.5
C1—C2—H2	120.1	H10B—C10—H10C	109.5
C3—C2—H2	120.1	O1X—S1X—N1X	111.3 (8)
O2—C3—C4	124.38 (16)	O1X—S1X—C7X	106.8 (8)
O2—C3—C2	114.58 (16)	N1X—S1X—C7X	97.7 (8)
C4—C3—C2	121.03 (17)	C1—N1X—S1X	127.0 (11)
C3—O2—C11	117.06 (16)	C1—N1X—H1X	116.5
O2—C11—H11A	109.5	S1X—N1X—H1X	116.5
O2—C11—H11B	109.5	C8X—C7X—C10X	112.9 (15)
H11A—C11—H11B	109.5	C8X—C7X—C9X	109.8 (14)
O2—C11—H11C	109.5	C10X—C7X—C9X	108.3 (16)
H11A—C11—H11C	109.5	C8X—C7X—S1X	107.4 (11)
H11B—C11—H11C	109.5	C10X—C7X—S1X	106.6 (14)
C5—C4—C3	117.97 (16)	C9X—C7X—S1X	111.8 (11)
C5—C4—H4	121.0	C7X—C8X—H8D	109.5
C3—C4—H4	121.0	C7X—C8X—H8E	109.5
C6—C5—C4	122.33 (17)	H8D—C8X—H8E	109.5
C6—C5—H5	118.8	C7X—C8X—H8F	109.5
C4—C5—H5	118.8	H8D—C8X—H8F	109.5
C5—C6—C1	119.20 (17)	H8E—C8X—H8F	109.5
C5—C6—H6	120.4	C7X—C9X—H9D	109.5
C1—C6—H6	120.4	C7X—C9X—H9E	109.5
C9—C7—C8	112.73 (17)	H9D—C9X—H9E	109.5
C9—C7—C10	111.27 (18)	C7X—C9X—H9F	109.5
C8—C7—C10	110.95 (19)	H9D—C9X—H9F	109.5
C9—C7—S1	111.54 (13)	H9E—C9X—H9F	109.5
C8—C7—S1	105.49 (13)	C7X—C10X—H10D	109.5
C10—C7—S1	104.40 (18)	C7X—C10X—H10E	109.5
C7—C8—H8A	109.5	H10D—C10X—H10E	109.5
C7—C8—H8B	109.5	C7X—C10X—H10F	109.5
H8A—C8—H8B	109.5	H10D—C10X—H10F	109.5
C7—C8—H8C	109.5	H10E—C10X—H10F	109.5
H8A—C8—H8C	109.5		
			
O1—S1—N1—C1	−113.14 (17)	N1X—C1—C6—C5	−152.9 (8)
C7—S1—N1—C1	136.38 (17)	O1—S1—C7—C9	−59.63 (15)
S1—N1—C1—C6	−26.5 (3)	N1—S1—C7—C9	52.62 (15)
S1—N1—C1—C2	154.05 (16)	O1—S1—C7—C8	177.66 (12)
S1—N1—C1—N1X	−104.9 (15)	N1—S1—C7—C8	−70.09 (14)
C6—C1—C2—C3	−2.1 (3)	O1—S1—C7—C10	60.64 (17)
N1—C1—C2—C3	177.31 (18)	N1—S1—C7—C10	172.89 (16)
N1X—C1—C2—C3	149.0 (8)	C6—C1—N1X—S1X	−10.0 (16)
C1—C2—C3—O2	−177.99 (17)	C2—C1—N1X—S1X	−162.5 (9)
C1—C2—C3—C4	3.4 (3)	N1—C1—N1X—S1X	105 (2)
C4—C3—O2—C11	1.7 (3)	O1X—S1X—N1X—C1	125.7 (13)
C2—C3—O2—C11	−176.92 (19)	C7X—S1X—N1X—C1	−122.9 (14)
O2—C3—C4—C5	179.45 (19)	O1X—S1X—C7X—C8X	−178.2 (12)
C2—C3—C4—C5	−2.1 (3)	N1X—S1X—C7X—C8X	66.7 (13)
C3—C4—C5—C6	−0.5 (3)	O1X—S1X—C7X—C10X	−57.0 (15)
C4—C5—C6—C1	1.6 (3)	N1X—S1X—C7X—C10X	−172.0 (15)
C2—C1—C6—C5	−0.3 (3)	O1X—S1X—C7X—C9X	61.2 (14)
N1—C1—C6—C5	−179.7 (2)	N1X—S1X—C7X—C9X	−53.9 (14)

### Hydrogen-bond geometry (Å, °)

**Table:**

*D*—H···*A*	*D*—H	H···*A*	*D*···*A*	*D*—H···*A*
N1—H1···O1^i^	0.88	2.24	2.884 (2)	130
N1X—H1X···O1X^ii^	0.88	2.21	2.94 (2)	141

Symmetry codes: (i) −*x*+1, −*y*, −*z*+1; (ii) −*x*+1, −*y*+1, −*z*+1.
